# Statistical Analysis and Optimization of the Brilliant Red HE-3B Dye Biosorption onto a Biosorbent Based on Residual Biomass

**DOI:** 10.3390/ma15207180

**Published:** 2022-10-14

**Authors:** Gabriel Dan Suditu, Alexandra Cristina Blaga, Ramona-Elena Tataru-Farmus, Carmen Zaharia, Daniela Suteu

**Affiliations:** 1 Department of Chemical Engineering, “Cristofor Simionescu” Faculty of Chemical Engineering and Environmental Protection, “Gheorghe Asachi” Technical University of Iasi, D. Mangeron Blvd., No. 73A, 700050 Iasi, Romania; 2 Department of Organic, Biochemical and Food Engineering, “Cristofor Simionescu” Faculty of Chemical Engineering and Environmental Protection, “Gheorghe Asachi” Technical University of Iasi, D. Mangeron Blvd., No. 73A, 700050 Iasi, Romania; 3 Department of Environmental Engineering and Management, “Cristofor Simionescu” Faculty of Chemical Engineering and Environmental Protection, “Gheorghe Asachi” Technical University of Iasi, D. Mangeron Blvd., No. 73A, 700050 Iasi, Romania

**Keywords:** biosorption, polysaccharides as biosorbent, organic dye, statistical analysis and optimization

## Abstract

Using various techniques, natural polymers can be successfully used as a matrix to immobilize a residual microbial biomass in a form that is easy to handle, namely biosorbents, and which is capable of retaining chemical species from polluted aqueous media. The biosorption process of reactive Brilliant Red HE-3B dye on a new type of biosorbent, based on a residual microbial biomass of *Saccharomyces pastorianus* immobilized in sodium alginate, was studied using mathematical modeling of experimental data obtained under certain conditions. Different methods, such as computer-assisted statistical analysis, were applied, considering all independent and dependent variables involved in the reactive dye biosorption process. The optimal values achieved were compared, and the experimental data supported the possibility of using the immobilized residual biomass as a biosorbent for the studied reference dye. The results were sufficient to perform dye removals higher than 70–85% in an aqueous solution containing around 45–50 mg/L of reactive dye, and working with more than 20–22 g/L of prepared immobilized microbial biosorbent for more than 9.5–10 h. Furthermore, the proposed models agreed with the experimental data and permitted the prediction of the dye biosorption behavior in the experimental variation field of each independent variable.

## 1. Introduction

Water is essential for the development and survival of all living species. Thus, there is a growing concern about finding new drinking water sources, as well as ensuring the pollution prevention/remediation of existing water resources and the efficient decontamination of wastewater directly discharged into natural resources.

Dyes represent a category of persistent organic micropollutants whose presence visually affects the water luster and the degradation products that can be obtained. For these reasons, many methods of removing them have been identified, including coagulation–flocculation, ion exchange using synthetic exchangers, advanced oxidation and reduction, and adsorption on various synthetic or natural materials [1].

Although these methods have a high separation efficiency, they also have disadvantages, such as high operating costs, production of by-products to be capitalized/valorized, and limitations regarding the types and amounts of pollutants retained [1].

One of the most frequently used methods is adsorption, because it is cheap and allows the adsorptive material to be adapted to concrete working conditions.

Water pollution is a severe environmental and public problem. Therefore, different materials have been investigated for their ability to retain pollutants from aqueous environments, with a particular interest in developing cheap and effective adsorbents. One possibility investigated is associated with natural polymers, especially polysaccharides (chitin, starch, chitosan, cellulose, pectine, alginate, agarose, etc.), which are used because they offer several advantages: renewability, high abundance, biodegradability, eco-friendliness, economic feasibility, and high adsorption capacity [2,3,4]. Moreover, through chemical reactions, polysaccharides can produce macromolecular superstructures (nanoparticles, fibers, membranes, films, beads, and gels) that can be used as biosorbents for various pollutants’ removal from aqueous effluents.

Biosorption is a cost-effective technique for removing pollutants from wastewater using different biomass-based materials as adsorbents [4,5,6]. The process relies on the diffusion of pollutant species through the pores of the biosorbent and the physical or chemical interactions between the biomass functional groups and the adsorbate [7,8]. A vast array of biomaterials have been analyzed for their potential biosorptive properties: microbial biomass, and industrial and agricultural biomass (wood, fruit, plant or shells waste, containing cellulose, hemicellulose, pectin, and lignin), in the free form [9,10], or immobilized, in order to improve the biosorption performance [4]. The process is usually analyzed using single or multi-component equilibrium models (Langmuir, Freundlich, Dubinin—Radushkevich, Temkin isotherms), adsorption kinetics, and controlling mechanism models, which validate the experimental data, to elucidate the biosorption phenomenon in a bulk phase [11,12]. Different models and optimization algorithms have been applied to analyze and estimate biosorbent capacity (q) and biosorption efficiency (R, %). For commercial/industrial exploitation of biosorption, it is crucial to obtain efficient experimental models for biosorption process control and regulation, suitable for large-scale setups [10,11,12,13,14,15]. Moreover, it is essential to improve the technological and mathematical optimization [14], after the active mechanism, involved interactions (adsorbate-biosorbent), and biosorption isotherm, kinetic, and thermodynamic properties have been understood and validated. Statistical analysis and optimization are commonly used in adsorption studies for mathematical process modelling and optimizing performance. Thus, the previously reported processes include the following: removal of Cr(VI) from the aqueous solutions via teff straw-based activated carbon [15]; biosorption of Acid Yellow and Acid Blue onto biomass obtained from brewery industrial waste/ spent brewery grains [16]; Cu^2+^ biosorption using *Oenococcus oeni* PSU1 [17]; Zn^2+^ biosorption using *Spirilina platensis* [18]; Cd^2+^ biosorption using *Turbinaria ornata* [12]; and Methylene Blue onto de-oiled algal biomass [19], among others. In this study, the removal of a reference model of reactive azo dye (Brilliant Red HE-3B) from aqueous colored solutions onto a newly prepared biosorbent, based on a residual immobilized biomass (with residual *Saccharomyces pastorianus* byproducts), is reported. A previous scientific report described this biosorbent, characterized by advanced analysis (EDX, SEM, FTIR) [20]. 

Brilliant Red HE-3B (BRed) is generally used in textile and other industrial applications. Its biosorption using residual Saccharomyces pastorianus (*S. pastorianus*) yeast immobilized in sodium alginate as a biosorbent has been analyzed, and it was reported as having a biosorption capacity of approximately 222 mg/g [17]. The process is strongly influenced by several operating factors: temperature, pH, S/L phases contact time (biosorption time), biosorbent and adsorbate concentration, and other particular characteristics. Moreover, a modeling and optimization procedure is beneficial for selecting the best process operating conditions for practical applications. In addition, this could provide the scientific basis for scaling-up biosorption, its control, and regulation, to ensure adequate results. 

Therefore, the dye biosorption process was studied using practical experiments and data analysis, considering three important influencing variables (biosorbent concentration, dye concentration, and biosorption contact time). These variables were established as significant for reactive dye biosorption in previously published reports [20,21] related to the analysis of specific adsorption isotherms, thermodynamic and kinetic models in association with the predicted biosorption mechanism, and its rate of control. Moreover, the prepared biosorbent material was physico-chemically characterized before and after the biosorption process of reactive Brilliant Red HE-3B dye, to underline its biosorption performance, using advanced analysis methods (SEM, FTIR, EDX), as reported in a previously published report [20].

This paper aimed to obtain viable experimental models for the biosorptive behavior of a residual microbial biomass of *S. pastorianus* immobilized in sodium alginate toward the selected dye. The modelling procedure used was the response surface method (RSM).

RSM, which is based on central composite design, is one of the traditional optimization methodologies and used in many chemical and biochemical research studies. It was first proposed in 1957 by Box and Hunter [22]. 

Operational aspects can be optimized using statistical approaches such as RSM to maximize a given process. For example, the RSM, with a minimal design of experiments (DOE), is now routinely employed for formulation optimization. In contrast to conventional procedures, statistical techniques can be utilized to ascertain how process factors interact.

The fundamental premise of RSM is to use a series of pre-planned experiments to determine the best response. Box and Wilson [23] suggested using a second-degree polynomial model for this. They noted that this model is only an approximation, but they adopted it because it allows easy estimation and application, even when little is known about the process.

The current biosorption process of the studied BRed reactive dye onto residual immobilized biomass was mathematically modelled by considering specific variation domains of three important independent process variables. The biosorption performance in retaining dye from aqueous solutions (%) was considered the dependent variable in the selected modeling strategy. Different experimental modeling methods can be applied in this sense, but we performed a statistical analysis assisted by a computer to find the optimum values. The possibility of using residual immobilized biomass as a biosorbent for retaining the studied reference reactive azo dye was supported by the experimental results, and through a computer assisted statistical analysis, the proposed model was found adequate for practical controlled biosorption applications.

## 2. Materials and Methods

### 2.1. Materials

Biomass. The microorganism used for biosorption were *Saccharomyces pastorianus* (Saccharomycetaceae family) [17], in the form of residual biomass after the brewing process and was provided by a local brewing company (Albrau, Onesti, Romania). Unicellular fungi *Saccharomyces pastorianus* is a by-product of the brewing industry are thus available in large quantities and is known for its capacity to enzymatically convert sugar into carbon dioxide and alcohol. The N-linked type mannoproteins, which make up the majority of yeast mannoproteins, contribute to the ionic properties of the yeast cell surface and can be used as biosorption functional groups. The residual *S. pastorianus* biomass is separated by centrifugation (8000 rpm), dried at 80 °C (until moisture is 2%), and then immobilized in sodium alginate.

Biosorbent. The biosorbent used in the experimental biosorption studies was prepared by immobilizing residual biomass (*S. pastorianus*) on sodium alginate using a simple dropping technique, performed using the synthesis methodology presented in a previously published report [20].

**Figure 1:**
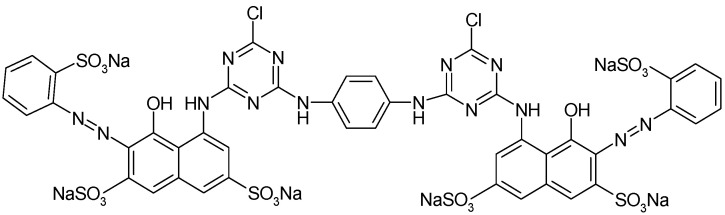
Chemical structure of reactive Brilliant Red HE-3B dye—CI 25810.

Adsorbate. A reactive dye, Brilliant Red HE-3B (BRed) (MW = 1430 g/mol, λ_max_ = 530 nm, from Bezema) with the chemical structure shown in Figure 1, was selected as a polluting chemical species (reference model of reactive azo dye) of an aqueous system for this study.

The stock solution (with an initial concentration of about 500 mg dye/L) was prepared from the commercial form of the dye powder. The working solutions were prepared using the corresponding dilution of the initial dye solution with distilled water.

### 2.2. Experimental Methods 

#### 2.2.1. Batch Biosorption Method

Experimental biosorption studies were conducted by mixing, in 50-mL Erlenmeyer flasks, different amounts of biosorbent with 25 mL of dye solution with a 0.1 mg/mL concentration. The biosorbent was obtained by immobilization in sodium alginate of a 5 percent dry matter (d.w.), represented by a residual microbial biomass of *S. pastorianus* (according to the specific experimental planning matrix). The residual biomass was pre-washed with distilled water, to remove traces of calcium chloride solution, which can cause dye precipitation. The pH values were adjusted with 0.1 N HCl solution (0.25 mL HCl 0.1 N/25 mL of dye solution) to the reported favorable value of pH = 3 (measured with a portable Hanna Instruments pH-meter) for BRed dye biosorption [20], and the temperature was maintained at approximately 25 °C. The contact time between the solid and liquid phases varied between 2.64 and 9.36 h (for a daily exchange at work, meaning between 158–562 min). The BRed dye concentration in the aqueous solution was determined spectrophotometrically using a Metertech SP-830 Plus spectrophotometer (Metertech Inc., New Taipei, Taiwan, version 1.06), at the dye’s maximum wavelength of 530 nm, following the Lambert–Beer law and its calibration curve.

The amount of dye removed from the aqueous solution was expressed by the amount of dye retained per gram of biosorbent, *q*, using relation (1). The efficiency of the dye biosorption was expressed by the degree of dye retention from the aqueous solution (R, or Y, %) using relation (2):

(1) $$q=\frac{C_{0}-C}{G}\times V$$

(2) $$R\;\left(or\;Y\right)=\frac{C_{0}-C_{t}}{C_{0}}\times 100$$

where *C*_0_, *C*, and *C_t_* are the initial, residual, and at *t* biosorption time dye concentrations in the aqueous solution (mg/L); *G* is the amount of biosorbent (g), and *V* is the volume of aqueous solution (L).

#### 2.2.2. Biosorption Process Modeling Using Computer-Assisted Statistical Analysis

Studying and determining the ideal conditions for Brilliant Red HE-3B dye biosorption onto a biosorbent based on residual biomass was one of the objectives of the current investigation. This was accomplished through the use of a three-step process: (1) experiments (where an experimental plan was established and followed), (2) modeling (where data gathered in the preceding step were statistically modelled to determine a set of mathematical relations that could describe the processes; RSM represents the procedure used to complete this step), and (3) optimization (where the previously determined model, in combination with an optimizer, was used to identify the optimal process parameters).

Computer-assisted data analysis packages are increasingly being used for statistical data processing [18,19,24,25]. The advantage of this software is that it allows for much more efficient work processing, which leads to significant time savings and helps in obtaining more in-depth information on data, which might otherwise be lost.

The response surface technique algorithm was implemented using the MINITAB program from the Minitab Institute in the United States. The experimental results were used to generate equations describing the relationships between selected process parameters and model responses. The optimum conditions for dye biosorption were established following the relationships between sorption yield and the independent variables chosen (residual immobilized biomass, dye concentration, and biosorption time).

MINITAB is a computer-assisted data analysis software that provides information about quality data sets, to improve processes. Based on the mathematical models provided by the program, valuable information can be discovered, which allows the optimization of the analyzed biosorption process. 

The ‘design of experiment’ methodology allows constructing a mathematical model of a process by performing a minimum number of experiments. The model thus obtained provides the connections between the independent process operating variables, to optimize the process studied.

In all our biosorption experiments on a residual immobilized biomass applied for reactive BRed dye removal, the following independent biosorption variables were considered: the immobilized biosorbent concentration (*Z*_1_, g/L), BRed dye concentration (*Z*_2_, mg/L), and biosorption contact time (*Z*_3_, h). The dye removal from aqueous solution (Y, or R, %) was chosen as an optimization criterion, or decision function. The experimental data were used according to different planning matrices: two had been used in a previous report [21], and the other three were distinct to this work. An appropriate analysis of variance was carried out for the model validation. 

For experimental data analysis and interpretation, the MINITAB software (version 17.1.0, Minitab, Ltd., Coventry, UK) package was used to test three models: linear, full quadratic, and cubic, considering the real values of all selected independent process variables, i.e., Y = f(Z_1_, Z_2_, Z_3_).

## 3. Results and Discussion

### 3.1. Biosorption Process Performance

Considering the conclusions of our previous studies regarding the factors influencing the reactive Brilliant Red-HEB 3B dye biosorption process, onto a residual biomass of *S. pastorianus* immobilized in sodium alginate [20], the operating conditions for obtaining significant dye removal efficiencies and the highest BRed dye biosorption capacities (i.e., *q* > 80–100 mg of dye/g of immobilized biosorbent) should be pH 3, a temperature of 25–30 °C, with a biosorbent concentration of at least 2.60 g/L (with 5% d.w.), depending on the diameter of the biomass-based granules (in this case study, around 2 mm), and a contact time of S/L phases in dye biosorption of at least 7.4 h, up to 10 h (exchange at work or more), for dye concentrations in an aqueous solution in the range of 16.88–174.08 mg/L. Our previous report [20] concluded that the biosorption of BRed dye on the tested immobilized biosorbent was efficient for small diameter granules (high biosorption capacity values were found), regardless of the biosorbent dose. In this case, a large contact surface is ensured, and a beneficial contact between the dye molecules and the immobilized biomass active sites favors the diffusion process. Moreover, using the determined biosorption energy value (E) (i.e., 8.28–11.18 KJ/mol) [20], it was proposed that the mechanism of this dye biosorption process is based on physical bonding, involving van der Waals interactions, hydrogen, dipole–dipole interactions, and electrostatic attraction between the positively charged surface sites of the immobilized biosorbent and the functional groups of the reactive BRed dye.

**Figure 2:**
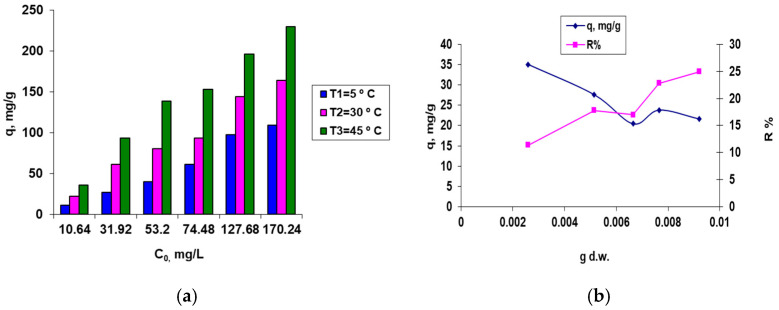
The influence of the initial dye concentration and solution temperature (**a**) and the quantity of biosorbent per 25 mL aqueous dye solution (**b**) on the biosorption of Brilliant Red HE-3B dye onto the residual microbial biomass of *S. pastorianus* immobilized in sodium alginate. Conditions: pH = 3; T = 25 °C; contact time = 24 h; 2.6 g/L biosorbent (**a**) and *C*_0_ = 31.95 mg/L (**b**).

The influence of certain selected independent variables (influencing factors such as the initial dye concentration in the aqueous solution and the biosorbent concentration) on the biosorption capacity of the residual immobilized biomass (*q*, mg/g), and/or the biosorption efficiency of the dye retention onto the prepared immobilized biosorbent from aqueous solution (R, or Y, %), was studied and the new data are summarized in Figure 2. 

In the selected experimental data (Figure 2), the biosorption results after a biosorption period of 24 h were considered and always attained a biosorption equilibrium, implying a static regime favorable for a discontinuous operating/working regime. In the present research work, which processed the experimental data using statistical analysis for modeling and identification of optimum conditions, the biosorption results were assessed for a biosorption period of no more than 10 h, using a continuous operating regime. The primary objective was to achieve a high BRed dye biosorption efficiency on the residual immobilized microbial biomass (>70–85 percent) and to estimate the maximum dye removal value, in conjunction with the specific optimal operating conditions necessary to achieve this, namely the optimal biosorbent, initial dye concentration, and biosorption time. 

The results showed that BRed dye concentrations between 45 mg/L and 170 mg/L led to higher BRed dye removal rates of between 65.00% and 85.41%.

As shown in Figure 2a, the highest dye biosorption capacities (*q*) onto residual immobilized biomass were obtained at the highest tested temperature (T_3_ = 45 °C), working with a high initial dye concentration in the aqueous solution. For room temperature (around 25 °C, close to the T_2_ temperature) working at pH 3 with an initial BRed dye concentration between 31.92–53.2 mg/L, the biosorption capacity of the prepared biosorbent based on residual immobilized biomass was in the range of 60–80 mg/g, working with a biosorbent concentration of around 2.60 g/L (with 5% d.w.). For an initial BRed dye concentration of 31.95 mg/L in the aqueous solution (Figure 2b), the dye removal percentage from the aqueous solution was around 24.91%, corresponding to a BRed dye biosorption capacity of residual immobilized biomass of around 35.05 mg/g. Better results were obtained for higher reactive BRed dye concentrations in the aqueous system, and when working with greater biosorbent concentrations in the same operating conditions of pH 3 and T = 25 °C (to avoid residual biomass degradation).

### 3.2. Experimental Modeling Using Computer-Assisted Statistical Analysis

An example of a specific experimental planning matrix used in modeling the reactive BRed dye biosorption onto the residual immobilized microbial biomass is presented in Table 1.

The three independent variables taken into account for the process modeling and subsequent optimization, their variation interval, and coding levels are presented in Table 2.

The experimental results were analyzed and interpreted using the MINITAB 17.1.0 software package. Three models were tested: linear, full quadratic, and cubic. The statistical results for all tested models are presented in Table 3.

For the operating process parameters that were considered, a full second-order polynomial model was generated using multiple regression techniques. Relation 3 is a representation of the regression equation for uncoded variables.

A full second-order polynomial model was obtained using multiple regression techniques for the considered operating process parameters. The regression equation is represented by the relation (3):

Y = −94.1 + 2.157 × Z_1_ + 104.1 × Z_2_ + 15.97 × Z_3_ − 0.01259 × Z_1_ × Z_1_ − 118.2 × Z_2_ × Z_2_ − 0.434 × Z_3_ × Z_3_ + 0.052 × Z_1_ × Z_2_ − 0.1166 × Z_1_ × Z_3_ + 4.45 × Z_2_ × Z_3_ (3)

Analyzing the *p*-value showed that a few terms were not significant, and they were removed using the backward elimination of terms method (BET). 

Regression Equation. The simplified regression equation established with the statistical results presented in the previous table (Table 1) was as follows (relation (4)):

Y = −86.8 + 2.155 Z_1_ + 131.6 Z_2_ + 11.95 Z_3_ − 0.01241 Z_1_ × Z_1_ − 115.1 Z_2_ × Z_2_ − 0.1166 Z_1_ × Z_3_ (4)

There was a slight decrease in R^2^ compared to the original model but an improvement in the values of PRESS and R-sq (pred). Table 4 presents the variance analysis results, from which each term’s contribution can be observed. The highest contribution was from the biosorption contact time (Z_3_), followed by the dye concentration (Z_2_) and the biosorption concentration (Z_1_), as well as the association between the biosorption contact time and the biosorbent concentration (Z_1_×Z_3_), to perform the highest BRed dye removal from the aqueous solution.

**Figure 3:**
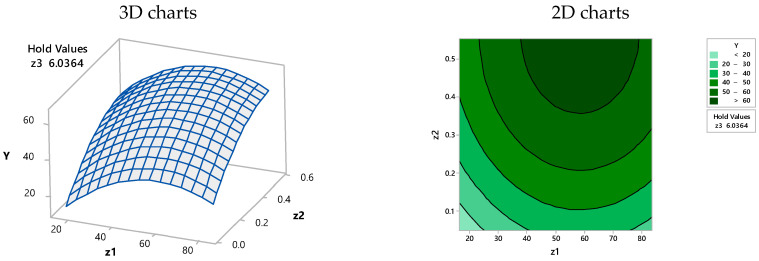
Surface plot (**left**) and contour plot (**right**)—efficiency (Y) vs. biosorbent concentration (Z_1_) and BRed dye concentration (Z_2_) at a constant biosorption contact time (Z_3_ = 6.0364 h).

**Figure 4:**
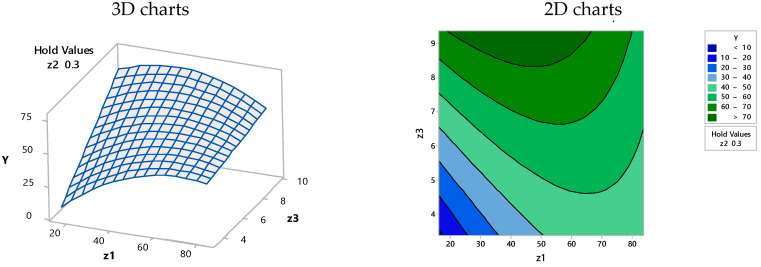
Surface plot (**left**) and contour plot (**right**)—efficiency (Y) vs. biosorbent concentration (Z_1_) and biosorption contact time (Z_3_) at a constant BRed dye concentration (Z_2_ = 0.3 mg/L).

**Figure 5:**
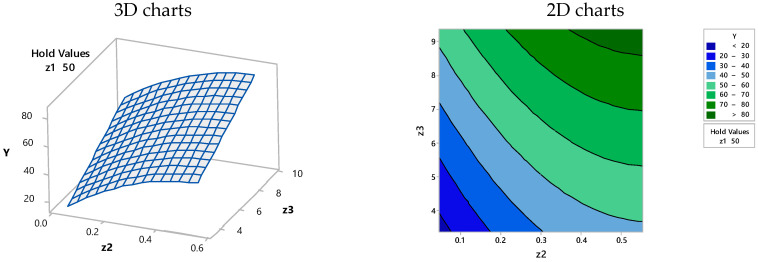
Surface plot (**left**) and contour plot (**right**)—efficiency (Y) vs. BRed dye concentration (Z_2_) and biosorption contact time (Z_3_) at a constant biosorbent concentration (Z_1_ = 50 g/L).

Based on the simplified Equation (4), and according to the MINITAB 17.1.0 model, the 2D and 3D representations from Figure 3, Figure 4 and Figure 5 were obtained, where the efficiency of dye retention was related to the two-functional operating variables under the condition of the third one that was kept constant.

### 3.3. Optimization of the Proposed Model Using Computer-Assisted Statistical Analysis

After applying the simplified model optimization process, obtained with the help of the MINITAB 17.1.0 program, five sets of optimal values were generated (Table 5). The first set predicted a yield of 85.4% when 42.864 g/L biosorbent is used to eliminate an amount of 0.55 mg of dye from 25 mL aqueous solution (22.10 mg dye/L), after 9.36 h. 

**Figure 6:**
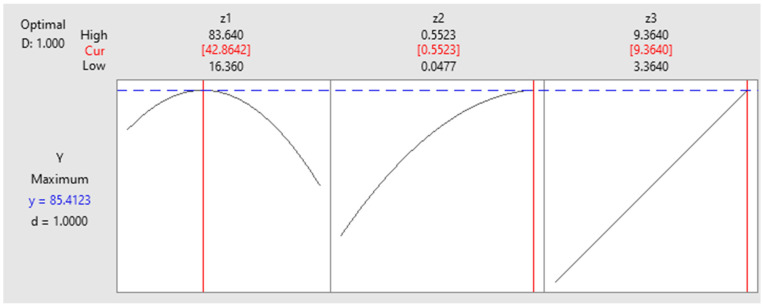
The maximum yield that can be obtained after the optimization of the biosorption process of reactive Brilliant Red HE-3B dye onto a residual *S. pastorianus* microbial biomass.

Figure 6 shows the maximum yield obtained after optimizing the studied biosorption process.

The optimum solutions indicated that the maximum dye biosorption removal was between 72.84% and 85.41%. The operating conditions for these results were: (1) biosorbent range, 19.23–42.86 g/L; (2) contact time, 8.92–9.3 h; and (3) initial dye concentration, 0.3–0.55 mg/L. This indicates that time has a large influence on the process, with the optimization having good removal rates when this parameter was extrapolated in reference to the experimental range. This influence could also be seen in the ANOVA analysis of the statistical model, where time had a 46.02% contribution.

## 4. Conclusions

Biosorption onto a residual biomass of *S. pastorianus* immobilized in sodium alginate can be applied with good efficiencies to reactive BRed dye removal from aqueous solutions. 

A mathematical model was proposed considering the biosorbent concentration (Z_1_), dye concentration (Z_2_), and biosorption time (Z_3_) as independent process variables, and the BRed dye removal as the decision function or optimization criterion (Y, %). The maximal values of all variables for the proposed optimization criterion were determined and evaluated in association with the significance/importance of each variable. 

Five optimum solutions were proposed using MINITAB 17.1.0 computational modeling, which recommended a dye biosorption efficiency of 72.85–85.41%, working with an immobilized biosorbent concentration of 19.23–50.00 g/L, and a duration of between 8.92–9.35 h, for a BRed dye concentration in an aqueous solution of 29–55 mg/L. 

These maximum solutions are encouraging (BRed reactive dye removal >72.84%), and thus this biosorption setup can continue at a larger scale, with other potential process variable improvements for programmed (controlled) process optimization.

## Figures and Tables

**Table 1:** Example of the experimental planning matrix used in the statistical analysis.

Exp. No.	Z_1_, (g/L)	Z_2_, (mg/L)	Z_3_, (h)	Y_ei_ (%)
1	6	30	4	13.62
2	18	30	4	34.79
3	6	70	4	33.35
4	18	70	4	48.49
5	6	30	8	52.63
6	18	30	8	72.49
7	6	70	8	47.06
8	18	70	8	74.18
9	1.908	50	6	36.68
10	22.09	50	6	63.32
11	12	16.36	6	34.65
12	12	83.64	6	51.91
13	12	50	2.64	44.94
14	12	50	9.36	69.66
15	12	50	6	56.74
16	12	50	6	55.38
17	0.60	50	6	56.82
18	0.60	50	6	58.11
19	0.60	50	6	54.90
20	0.60	50	6	56.50

**Table 2:** The coding level of the variables and the actual values used to design the experiments.

Variable	Symbol	Coding Level Values				
		−α	−1 (Lower Level)	0 (Base Level)	+1 (Higher Level)	+α
Residual immobilized biomass, (g/L)	Z_1_	1.91	6	12	18	22.09
Dye concentration, (mg/L)	Z_2_	16.36	30	50	70	83.64
Biosorption time, (h)	Z_3_	2.64	4	6	8	9.36

**Table 3:** Model summary of analysis using MINITAB 17.1.0 software.

Model	S	R-sq	R-sq(adj)	PRESS	R-sq(pred)
Linear	7.10238	80.72%	77.10%	1318.14	68.51%
Full quadratic	4.00812	96.16%	92.71%	1079.82	74.21%
Cubic	1.14226	99.84%	99.41%	-	-
Full quadratic simplified	3.99308	95.05%	92.76%	609.094	85.45%

Where [26]: S denotes how far the data values deviate from the fitted values. S is measured in response units; R-sq is the percentage of variation in response explained by the model. This is calculated as 1 minus the ratio of the error sum of squares (the variation that the model does not explain) to the overall sum of squares (the entire variation in the model); R-sq(adj) is the proportion of response variance explained by the model, adjusted for the number of predictors in the model relative to the number of observations. Adjusted R^2^ is determined as 1 minus the mean square error (MSE) to mean square total ratio (MS Total). PRESS is the prediction error sum of squares, which determines the difference between the fitted and observed values. The sum of squares of the residual error (SSE), which is the sum of the squared residuals, is comparable to PRESS. However, PRESS calculates the residuals differently. R-sq(pred) is obtained by systematically deleting each observation from the data set, estimating the regression equation, and measuring how well the model predicts the deleted observation.

**Table 4:** Analysis of Variance.

Source	DF	Seq SS	Contribution	Adj SS	Adj MS	F-Value	*p*-Value
Model	6	3978.92	95.05%	3978.92	663.15	41.59	0.000
Linear	3	3379.10	80.72%	3233.23	1077.74	67.59	0.000
Z_1_	1	251.25	6.00%	153.01	153.01	9.60	0.008
Z_2_	1	1201.35	28.70%	1201.35	1201.35	75.35	0.000
Z_3_	1	1926.49	46.02%	1878.87	1878.87	117.84	0.000
Square	2	425.88	10.17%	425.88	212.94	13.36	0.001
Z_1_×Z_1_	1	328.29	7.84%	358.76	358.76	22.50	0.000
Z_2_×Z_2_	1	97.59	2.33%	97.59	97.59	6.12	0.028
2-Way Interaction	1	173.94	4.15%	173.94	173.94	10.91	0.006
Z_1_×Z_3_	1	173.94	4.15%	173.94	173.94	10.91	0.006

Where [27]: DF is the total degrees of freedom of the amount of information in the supplied data. This study used this information to estimate the values of unknown population parameters. The number of observations in the sample determined the total DF. The DF of a phrase indicates how much information it contains. Increasing the sample size yields more information about the population, increasing the overall DF. Conversely, increasing the number of terms in the model consumes more information, reducing the DF available for evaluating the variability of parameter estimations. Seq SS stands for the sequential sums of squares, which are measures of variation for various model components. The sequence in which the terms are entered into the model determines the sequential sums of squares. Contribution shows the percentage of the total sequential sums of squares that each source in the analysis of variance table contributes to (Seq SS). Adj SS signifies the adjusted sums of squares, which are variation measurements for various model constituents. The order of model predictors does not affect how the modified sum of squares is computed. Adj MS, the adjusted mean squares metric measures how much variation, given all other terms included in the model, and independently of their entry order, a term or a model explains. Unlike the adjusted sums of squares, the adjusted mean squares consider the degrees of freedom. The variation surrounding the fitted values is the adjusted mean square of the error, commonly known as MSE or s2. F-Value is a test statistic used to examine whether a phrase is connected with a response. Minitab calculates the *p*-value using the F-value, which is then used to determine the statistical significance of the terms and model. A sufficiently large F-value suggests that the term or model is noteworthy. *p*-Value: This probability gauges the strength of the evidence in opposition to the null hypothesis. Stronger evidence is presented against the null hypothesis via lower probabilities. The model accounts for variation in the answer if the *p*-value is lower than or equal to the significance level.

**Table 5:** Optimum solutions for maximum dye removal efficiency.

Solution	Z_1_ (g/L)	Z_2_/100 (mg/L)	Z_3_ (h)	Y Fit (%)	Composite Desirability
1	42.86	0.55	9.36	85.41	1.00000
2	50.00	0.30	9.01	74.18	1.00000
3	29.99	0.29	9.36	74.18	1.00000
4	19.23	0.55	8.92	74.17	0.99981
5	24.67	0.55	9.36	72.85	0.97775

## Data Availability

Data is contained within the article.

## References

[1] Zaharia C., Suteu D., Puzyn T., Mostrag-Szlichtyng A. (2012). Textile Organic Dyes–Characteristics, Polluting Effects and Separation/Elimination Procedures from Industrial Effluents—A Critical Overview. Organic Pollutants—Ten Years after the Stockholm Convention: Environmental and Analytical Update.

[2] Qi X., Tong X., Pan W., Zeng Q., You S., Shen J. (2021). Recent advances in polysaccharide-based adsorbents for wastewater treatment. J. Clean. Prod..

[3] Aravind J., Kamaraj M., Muthukumaran P., Thirumurugan A., Ramachandran K.K., Kalia S. (2021). Plant polysaccharides-based adsorbents. Natural Polymers-Based Green Adsorbents for Water Treatment.

[4] Blaga A.C., Zaharia C., Suteu D. (2021). Polysaccharides as Support for Microbial Biomass-Based Adsorbents with Applications in Removal of Heavy Metals and Dyes. Polymers.

[5] Pham V.H.T., Kim J., Chang S., Chung W. (2022). Bacterial Biosorbents, an Efficient Heavy Metals Green Clean-Up Strategy: Prospects, Challenges, and Opportunities. Microorganisms.

[6] Morales-Barrera L., Cristiani-Urbina E. (2022). Equilibrium Biosorption of Zn^2+^ and Ni^2+^ Ions from Monometallic and Bimetallic Solutions by Crab Shell Biomass. Processes.

[7] Madeła M., Skuza M. (2021). Towards a Circular Economy: Analysis of the Use of Biowaste as Biosorbent for the Removal of Heavy Metals. Energies.

[8] Blagojev N., Vasić V., Kukić D., Šćiban M., Prodanović J., Bera O. (2021). Modelling and efficiency evaluation of the continuous biosorption of Cu(II) and Cr(VI) from water by agricultural waste materials. J. Environ. Manag..

[9] Zaharia C. (2015). Application of waste materials as ‘low cost’ sorbents for industrial effluent treatment. A comparative overview. Int. J. Mater. Prod. Technol..

[10] Suteu D., Zaharia C., Blaga A.C., Zaharia C. (2012). Biosorption-current bioprocess for wastewater treatment (chapter 10). Current Topics, Concepts and Research Priorities in Environmental Chemistry.

[11] Hussein K.A., Hassan D.H., Joo J.H. (2011). Potential capacity of Beauveria bassiana and Matarhizium anisopliae in the biosorption of Cd^2+^ and Pb^2+^. J. Gen. Appl. Microbiol..

[12] Fawzy M.A., Darwish H., Alharthi S., Al-Zaban M.I., Noureldeen A., Hassan S.H.A. (2022). Process optimization and modeling of Cd ^2+^ biosorption onto the free and immobilized Turbinaria ornata using Box-Behnken experimental design. Sci. Rep..

[13] Fertu D.I., Dragoi E.N., Bulgariu L., Curteanu S., Gavrilescu M. (2022). Modeling the Biosorption Process of Heavy Metal Ions on Soybean-Based Low-Cost Biosorbents Using Artificial Neural Networks. Processes.

[14] Al-Zaban M.I., Alharbi N.K., Albarakaty F.M., Alharthi S., Hassan S.H.A., Fawzy M.A. (2022). Experimental Modeling Investigations on the Biosorption of Methyl Violet 2B Dye by the Brown Seaweed *Cystoseira tamariscifolia*. Sustainability.

[15] Beyan S.M., Prabhu S.V., Ambio T.A., Gomadurai C. (2022). A Statistical Modeling and Optimization for Cr(VI) Adsorption from Aqueous Media via Teff Straw-Based Activated Carbon: Isotherm, Kinetics, and Thermodynamic Studies. Adsorpt. Sci. Technol..

[16] Jaikumar V., Ramamurthi V. (2009). Statistical Analysis and Optimization of Acid Dye Biosorption by Brewery Waste Biomass Using Response Surface Methodology. Mod. Appl. Sci..

[17] El-Ahwany A.M.D. (2012). Statistical analysis and optimization of copper biosorption capability by Oenococcus oeni PSU-1African. J. Biotechnol..

[18] Alharbi N.K., Al-Zaban H.I., Alborakaty F.M., Abdelwahab S.F., Hassan S.H., Fawzy M.A. (2022). Kinetic, isotherm and thermodynamic aspects of Zn^2+^ biosorption by Spirulina platensis: Optimization of process variables of response surface methodology. Life.

[19] Maurya R., Ghosh T., Paliwal C., Shrivastav A., Chokshi K., Pancha I., Ghosh A., Mishra S. (2014). Biosorption of Methylene Blue by De-Oiled Algal Biomass: Equilibrium, Kinetics and Artificial Neural Network Modelling. PLoS ONE.

[20] Suteu D., Blaga A.C., Zaharia C., Cimpoesu R., Puițel A.C., Tataru-Farmus R.-E., Tanasă A.M. (2022). Polysaccharides Used in Biosorbents Preparation for Organic Dyes Retaining from Aqueous Media. Polymers.

[21] Zaharia C., Suteu D. (2022). Empirical Modeling and Optimization by Active Central Composite Rotatable Design: Brilliant Red HE-3B Dye Biosorption onto Residual Yeast Biomass-Based Biosorbents. Appl. Sci..

[22] Box G.E.P., Hunter J.S. (1957). Multi-Factor Experimental Designs for Exploring Response Surfaces. Ann. Math. Stat..

[23] Box G.E.P., Wilson K.B. (1951). On the Experimental Attainment of Optimum Conditions. J. R. Stat. Soc. Ser. B.

[24] Puițel A.C., Suditu G.D., Danu M., Ailiesei G.L., Nechita M.T. (2022). An Experimental Study on the Hot Alkali Extraction of Xylan-Based Hemicelluloses from Wheat Straw and Corn Stalks and Optimization Methods. Polymers.

[25] Nechita M.T., Suditu G.D., Puițel A.C., Drăgoi E.N. (2021). Differential evolution-based optimization of corn stalks black liquor decolorization using active carbon and TiO2/UV. Sci. Rep..

[26] Tripathi V.K., Ambekar S. (2019). Optimization and Analysis of Wear Rate of CFRP-NanoZno/Nanoclay Hybrid Composites Using RSM. J. Bio-Tribo-Corros..

[27] Meiabadi M.S.S.M., Kazerooni A., Moradi M., Torkamany M.J. (2020). Laser assisted joining of St12 to polycarbonate:Experimental study and numerical simulation. Optik.

